# Supplementary material for: Classification of flavors in cigarillos and little cigars and their variable cellular and acellular oxidative and cytotoxic responses
Source: PLoS One. 2019 Dec 11;14(12):e0226066. doi: 10.1371/journal.pone.0226066 (PMC6905550; doi:10.1371/journal.pone.0226066)
Supplement: Attachment — Submitted filename: Lawyer et al responses.docx [file pone.0226066.s002.docx]

**Manuscript ID:** PONE-D-19-21934

**Title:** Phylogenic classification of flavors in cigarillos and little cigars and their variable cellular and acellular oxidative and cytotoxic responses

**Authors:** Gina R Lawyer*, Monica Jackson*, Melanie Prinz, Thomas Lamb, Qixin, Wang, Thivanka Muthumalage, and Irfan Rahman

**Overall Response to Reviewers:**

We are very grateful for the time the editors and reviewers took to review this manuscript and provided their valuable comments/suggestions. We made sure to review our manuscript thoroughly and answer each question or suggestion that is posed.

**Comments and Responses:**

Reviewer #1:

***The manuscript by Jackson et al presented a novel aspect of phylogenic classification of flavors in cigarillos and little cigars and their variable cellular and acellular oxidative and cytotoxic responses. Flavored and flavorings in tobacco products are emerging. This is the first ever classification of flavors based on phylogeny.***

***The authors have also studied the oxidative stress and cellular responses by different flavors based on their phylogenic classifications. Most importantly, they showed different flavored cigars/cigarillos had variability in tested parameters between flavors as well as brands of the same flavor with their particle size and cellular toxicity. They found that fruits/candy and drinks/beverages were more toxic which are interesting.***

***Overall, this is the first study which attempted to classify flavors based on phylogeny. The authors must be commended for their efforts. This classification may be useful for other tobacco products including e-cigarette flavors. Experimental design with data rigor and reproducibility are paramount to this study with solid data presentation, innovative design, and outstanding nature of studies.***

***Cellular and acellular data presented which are directly correlated with the classified flavors. Particle sizes from flavored cigars are presented using a standard approach. The manuscript is well-written. However, the manuscript needs some careful data organization and analyses.***

Response: We are extremely thankful for the kind comments and recognition. We have made sure our best effort to review the data organization and analysis to improve this manuscript.

**Major Comments:** ***Table should contain more flavors based on the classified tree of flavors***

Response 1: We agree with the review’s comments, we also think more flavors based on our classification are needed and it’s really necessary for quantification and validation. Please check Figures 3 and 5, and Table 1, as suggested, we have ran several more cigars for ROS, and for particulate matter analyses in the revised manuscript.

**Major Comments 2: *Particulate matter concentrations from different flavored cigars based on classified flavor tree should be presented in detail in table 1.***

Response 2. We are thankful for this kind suggestion regarding better understanding of physical properties of different flavored cigars. As showing in our revised Table 1, different flavored cigars are added, and physical particular matter concentrations are shown in 4 different diameters with units of mg/m^3^ (see Table 1 and Figure 5).

**Major Comments 3*: Figure 2: should have the level of significance by more repeats and careful consideration of flavors. Figures 2B and C should contain statistical analyses. Fruits and beverages drinks should be highlighted with their data in this figure and other figures based on their conclusion.***

Response 3: We agree with the reviewer’s comments and concerns. We have carefully analyzed more acellular ROS using different flavored cigars, and analyze the statistical significance accordingly. As we mentioned in our discussion, due to the batch-batch variation of the commercial available cigars, part of results are still scattered. However, most of the cigars showed significant harmful effects even compared to research cigarette smoke.

We are really thankful for the suggestion of highlight the fruits/drinks flavored cigars. We have revised it accordingly in our results and discussion in the main text of the revised manuscript.

***Major Comments 4: Fig 3B, C should contain statistical analyses.***

Response 4: We thank the reviewer for their suggestion. We have applied statistical analysis accordingly to Figures 3b and 3c.

**Major Comments 5: *Fig 4: should contain control cigar/cigarillos like in other data figures.***

Response 5: We thank the reviewer for their suggestion. We have added a control of air to figure 4.

***Major Comment 6: Fig 5: more flavors should be studied based on classified flavors.***

Response 6: We thank the reviewer for their suggestion. More flavors have been tested for their particulate matter and acellular ROS. In Fig 3, more flavored cigars have been tested in ROS, several flavored cigars have been tested in this study. In table 1, several flavored cigars have been characterized with PM distribution and concentration; various flavored cigars have been measured.

***Reviewer #2:***

***The authors report phylogenetic classification of different flavors used in commercial cigars and cigarillos and their results on cellular and acellular oxidative and cytotoxic responses to these flavors in lung cell models. This is a novel study characterizing flavors using a novel strategy based on phylogenetic tree comparisons. Such analysis can be a prototype for testing other flavors like those for e-cigs. The study is based on logical measurements targeting ROS and cytotoxicity in relation to particulate size distribution. These data are striking in the sense that the flavors showed differential effects. This is the first report providing insights into and demonstrating health effects of flavor-infused cigars and cigarillos and may serve as a useful dataset for regulation of flavoring compounds.***

Response: We are extremely thankful for reviewer’s 2 encouraging comments and suggestions. We made sure to thoroughly address each suggestion or question.

***Major Comment 1: Table 1. More flavors need to be added for their characterization as listed by the authors.***

Response 1: We agree with the reviewer’s comments. We have addressed this comment previously. Please refer to the earlier response (reviewer #1, comment #1).

***Major Comments 2: Figure 1. The phylogenetic tree needs more clarity and bigger font size.***

Response 2: As suggested, we have increased the size of Figure 1 and the font within the figure so that it is clearer for readers.

***Major Comments 3: Figures 2 and 3. Need detailed information on vendors and brands of cigars and their ROS production.***

Response 3: We agree with the reviewer’s comments. We have the details that described the information of each cigar used with their brand, flavor based on packaging, and which category they have been included in the revised manuscript.

***Major Comment 4: Figure 4. Information on production of ROS by different portions of cigars may be meaningful.***

Response 4: We agree with the review’s comments. We have cut the cigar in different portion and measured the ROS generation. As showing in Figure 4, there is no significant difference among different portions.

***Major Comment 5. The authors should provide particulate matter data from other flavored cigars as listed in figures 1 and 6***.

Response 5: As suggested, more cigars have been tested and included into Figure 5 and table 1. We have addressed the similar comments (see above); please check the previous responses (reviewer #1, comments #6)

***Major Comment 6: Figure 6: How do Cytotoxicity data correlate with ROS data and particulate data***.

Response 6: We agree and understand reviewer’s comments and concerns. Generation of particulate matter affects the cellular uptake of chemicals or particles from cigar combustion, and it does participate in the cellular toxicity responses, as well as acellular ROS produced by cigars. However, there are more factors affects the cigars, such as the chemical compounds and deposition of cigar particles, all have cytotoxic influence. Though, we cannot fully connect the ROS and particulate matter data to our cell viability results, more detailed studies, such as cell toxicity test with different flavored chemicals, are in progress to figure out what is the key points caused cytotoxicity.

***Major Comment 7: The authors may pick key flavors and correlate them with ROS generation potential.***

Response 7: we thank for the reviewer’s comments/suggestions. We have picked up the key flavors that represent each flavoring category, as well as more cigars were used to test the ROS and particulate matter. Because of the cigars from different brands and even the different batch showed variations, there is no significantly specific trend across flavor and ROS. However, all the flavoring cigars showed higher ROS generation than research cigarette smoke.

***Additional notes from Reviewer #2: Additionally, the authors should fix the following: 1-Replace the term ‘phylogenic’ with the correct term which is ‘phylogenetic’. 2-Format the References list per the PLOSone format by cleaning up the extra information.***

Additional Notes Response: We thank the reviewer for his/her valuable suggestion, we have corrected and replaced accordingly.
