# Supplementary material for: Classification of flavors in cigarillos and little cigars and their variable cellular and acellular oxidative and cytotoxic responses
Source: PLoS One. 2019 Dec 11;14(12):e0226066. doi: 10.1371/journal.pone.0226066 (PMC6905550; doi:10.1371/journal.pone.0226066)
Supplement: Attachment — Submitted filename: Lawyer et al responses R2.docx [file pone.0226066.s003.docx]

**Manuscript ID:** PONE-D-19-21934R2

**Title:** Classification of flavors in cigarillos and little cigars and their variable cellular and acellular oxidative and cytotoxic responses

**Authors:** Gina R Lawyer*, Monica Jackson*, Melanie Prinz, Thomas Lamb, Qixin, Wang, Thivanka Muthumalage, and Irfan Rahman

**Academic Editor Comments:**

The Academic Editor has reviewed your revised manuscript and notes that the revisions have appropriately addressed the reviewers' concerns. However, the PLOS ONE staff editors have identified one aspect of the study methodology and some minor concerns with the text that we think should be addressed before we proceed. Please note that we have discussed these concerns with the Academic Editor, who agrees that these minor issues should be fixed. They are as follows:

**Specific Comments:**

1) We note the use of the T-REX web server and iTOL tool to develop a phylogenetic tree of cigarillo and little cigar flavorings. We are a concerned about the development of a “phylogenetic tree” in this setting, as phylogenetic trees are generally used to show evolutionary relationships, which is not what is reported in this paper. We would propose that this is instead referred to as a “classification”. This would involve several edits to the text, as follows:

- Title: Change to “Classification of flavors in cigarillos and little cigars and their variable cellular and acellular oxidative and cytotoxic responses”

**Response:** We have changed the title as suggested.

**Comment:**

- Abstract: Change “A new phylogenic classification system…” to “A new classification system…”

**Response:** This has been changed.

**Comment:**

- Introduction, p4: Delete the following sentences: “In biological classification, organisms are put into categories based on shared traits [20]. The closer organisms are in the tree, the more related they are to each other [20]. Although the amount of different cigar flavors is vast, the system used to classify these flavors must be able to convey a large amount of information clearly.”

**Response:** This has been amended.

**Comment:**

- Results p9: Change “To understand how the flavorings in cigars can be grouped a phylogenic tree was created” to “To understand how the flavorings in cigars can be grouped a new classification system was created”

**Response:** This has been changed.

**Comment:**

- Discussion p12: Change “Classification of flavoured cigars/cigarillos in a phylogenic tree should provide a convenient nomenclature/vocabulary in the scientific community as there was no scientific classification of cigar flavorings until the present” to “Classification of flavoured cigars/cigarillos should provide a convenient nomenclature/vocabulary in the scientific community as there was no scientific classification of cigar flavorings until the present”

**Response:** This has been changed.

**Comment:**

- Discussion p12: Change “The tree created allows for the categorization of flavors…” to “The classification system created allows for the categorization of flavors…”

**Response:** This has been changed.

**Comment:**

The tree presented in figure 1 should be changed to a different visual representation, for example a table showing to which flavour category and sub-category each flavour belongs. The legend to figure 1, as well as the Methods, should then be updated to remove the references to phylogenetic trees.

**Response:** We have changed the Figure 1, and legend is amended as suggested.

**Comment:**

2) We would like to suggest some changes to the text for clarity:

- Abstract: We note that none of the flavouring compounds tested in this work was as cytotoxic as cigarette smoke (in the Results, the authors state “Almost all cigars tested had a reduced in cell viability compared with the control, but were not more cytotoxic than smoke cigarette”). We think this finding should be added to the Abstract, for example “A differential cytotoxic response was observed with cigarillo smoke extract treatments: “fruits/candy” and “drinks” were the most toxic, but were not more cytotoxic than smoke from cigarettes.”

**Response:** This has been changed as suggested.

**Comment:**

- Discussion p12: The phrase “tobacco companies are creating more non-descriptive names to circumvent the regulation” may not be fully supported by the reference provided as the reference is from 2016 and that statement suggests that this practice is ongoing. We would suggest that this statement be removed; it should be sufficient to note that the introduction of additional flavourings will make comparison between categories more difficult.

**Response:** This has been changed as suggested

3) As you may know, PLOS ONE does not copyedit text before publication. Thus, please ensure that the text is copyedited for grammar and usage before the manuscript is resubmitted.

**Response:** we have now copyedited for grammar and usage in the revised manuscript.
